# Isolation of *Brucella microti* from Soil 

**DOI:** 10.3201/eid1408.080286

**Published:** 2008-08

**Authors:** Holger C. Scholz, Zdenek Hubalek, Jirina Nesvadbova, Herbert Tomaso, Gilles Vergnaud, Philippe Le Flèche, Adrian M. Whatmore, Sascha Al Dahouk, Monika Krüger, Csilla Lodri, Martin Pfeffer

**Affiliations:** * Bundeswehr Institute of Microbiology, Munich, Germany; † Academy of Sciences, Brno, Czech Republic; ‡ Centre d’Etudes du Bouchet, Vert le Petit, France; § Université Paris-Sud 11, Orsay, France; ¶ Veterinary Laboratories Agency, Addlestone, UK; # University of Aachen, Aachen, Germany; ** Veterinary University of Leipzig, Leipzig, Germany

**Keywords:** Brucella, Brucella microti, direct cultivation, environment, long-term survival, soil, letter

**To the Editor:** *Brucella microti* is a recently described *Brucella* species (*1*) that was isolated in 2000 from systemically infected common voles (*Microtus arvalis*) in South Moravia, Czech Republic. The organism is characterized by rapid growth on standard media and high metabolic activity, which is atypical for *Brucella* (*2*). The biochemical profile of *B*. *microti* is more similar to that of *Ochrobactrum* spp., of which most species are typical soil bacteria.

On the basis of the close phylogenetic relationship of *Brucella* spp. and *Ochrobactrum* spp. and the high metabolic activity of *B*. *microti*, we hypothesized that this *Brucella* species might also have a reservoir in soil. To test this hypothesis, we investigated 15 soil samples collected on December 11, 2007, from sites in the area where *B*. *microti* was isolated from common voles in 2000 (*2*). Ten of the samples were collected from the surface and at a depth of up to 5 cm near different mouse burrows 5 m apart. The remaining 5 samples were collected from an unaffected area without clinical cases of vole infection. The pH of soil samples ranged from 5.9 to 6.3. No frosts were recorded before the time of collection.

To specifically detect *B*. *microti* in soil samples, we have developed a PCR that targets a genomic island of 11 kb (H.C. Scholz et al., unpub. data) that is unique for *B*. *microti*. Briefly, primers Bmispec_f (5′-AGATACTGGAACATAGCCCG-3′) and Bmispec_r (5′-ATACTCAGGCAGGATACCGC-3′) were used to amplify a 510-bp fragment of the genomic island. PCR conditions were denaturation at 94°C for 5 min, followed by 29 cycles at 94°C for 30 s, 60°C for 30 s, and 72°C for 30 s. Total DNA was prepared from 0.5 g of each soil sample by using the MO BIO Ultra Clean Soil DNA Kit (Dianova, Hamburg, Germany). DNA was eluted with 50 μL of double-deionized water of which 2 μL was used in PCRs. Template DNA of *B*. *microti* CCM 4915^T^ was used as a positive control. Type strains of all recognized *Brucella* species, 1 strain of each biovar of all species, and type strains of 11 *Ochrobactrum* species were used as negative controls.

In this PCR, 5 of 15 soil samples and the positive control were positive for the 510-bp fragment; other *Brucella* spp. and *Ochrobactrum* spp. were negative. Of the 5 positive samples, 3 were collected from surface soil collected near mouse burrows. However, the remaining 2 positive samples were collected from the unaffected and supposedly negative-control area.

For direct cultivation of *Brucella* spp. from soil, 2 g each of 2 selected PCR-positive samples with the highest amplification rate (both from the affected area) were thoroughly homogenized in 5 mL of phosphate-buffered saline (PBS), pH 7.2, in 50-mL tubes. Of a serial dilution in PBS (10^0^–10^–4^), 100 μL was plated onto *Brucella* agar (Merck, Darmstadt, Germany) supplemented with 5% (vol/vol) sheep blood (Oxoid, Wesel, Germany) and *Brucella* selective supplement (Oxoid) and incubated at 37°C. Twenty suspicious colonies from the 10^0^ dilution plate of 1 soil sample were subcultivated on *Brucella* selective agar. Two of the subcultivated bacteria (BMS 17 and BMS 20) reacted positively with monospecific anti-*Brucella* (M) serum. Both isolates were positive in the *B*. *microti*–specific PCR. Sequencing of the 510-bp fragments from both strains (GenBank accession nos. AM943814 and AM943815) and comparison with the known nucleotide sequence of *B*. *microti* showed 100% identity.

To confirm that strains BMS 17 and BMS 20 were *B*. *microti*, these strains were subjected to multilocus sequence analysis and multilocus variable number of tandem repeat analysis (MLVA) as described (*1**,**3**–**5*). Multilocus sequence typing profiles of these strains were identical to the type strain *B*. *microti* CCM 4915^T^ and strain CCM 4916. MLVA showed that these strains also clustered with *B*. *microti* strains CCM 4915^T^ and CCM 4916, with identical panel 1 and panel 2A genotypes but a different panel 2B genotype.

In summary, we successfully isolated *B*. *microti* from soil samples collected at the same site 7 years after primary isolation of this novel species from common voles. *B*. *microti* could still be isolated from the same soil samples 6 months after storage at 4°C. This finding indicates long-term survival of *B*. *microti* in soil; thus, soil might function as a reservoir of infection. Identification of *B*. *microti* as a potential soil bacterium is consistent with *Brucella* spp. whole genome sequencing data, in particular with the genome sequence of *B*. *suis*, which exhibits fundamental similarities with plant pathogens such as *Agrobacterium* spp. and *Rhizobium* spp. (*6*). Whether soil is the primary habitat of *B*. *microti* or other vectors, such as nematodes, remains to be investigated.
